# Shrinkage of a Giant Type-B Dissecting Aneurysm Treated by Complete False Lumen Occlusion 20 Years after Presentation: A Case Report

**DOI:** 10.3400/avd.cr.21-00008

**Published:** 2021-06-25

**Authors:** Akimasa Morisaki, Etsuji Sohgawa, Yosuke Takahashi, Hiromichi Fujii, Yoshito Sakon, Noriaki Kishimoto, Kokoro Yamane, Toshihiko Shibata

**Affiliations:** 1 Department of Cardiovascular Surgery, Osaka City University Graduate School of Medicine, Osaka, Osaka, Japan; 2 Department of Diagnosis and Interventional Radiology, Osaka City University Graduate School of Medicine, Osaka, Osaka, Japan

**Keywords:** endovascular repair, false lumen occlusion, type-B aortic dissecting aneurysm

## Abstract

In this study, we report the case of a 47-year-old female who presented with extensive acute type IIIb aortic dissection and cerebral infarction. At 69 years of age, dilatation of the descending aorta was noted to be more than 70 mm with compression of the left atrium. We performed endovascular repair with distal false lumen occlusion. However, further dilatation of the descending aorta with false lumen flow from the re-entry of the common carotid artery was detected. She subsequently underwent additional proximal false lumen occlusion by embolization at the aortic arch. A year later, as per her computed tomography angiography findings, appreciable shrinkage of the descending aorta without endoleakage was observed.

## Introduction

Recently, endovascular intervention has been recommended as the first-line treatment for acute or subacute complicated type-B aortic dissection because of its favorable results with good aortic remodeling.^1)^ In contrast, endovascular intervention for chronic type-B dissecting aortic aneurysm remains controversial due to its higher late re-intervention rates compared with surgical intervention. However, endovascular intervention has been determined to result in lower early mortality and morbidity rates compared with open surgery.^2,3)^ In this study, we report on a patient with a successful outcome (significant shrinkage of the aneurysm) after undergoing endovascular repair with complete false lumen occlusion more than 20 years after she was diagnosed with type-B dissecting giant aneurysm.

## Case Report

**Figure 1:**
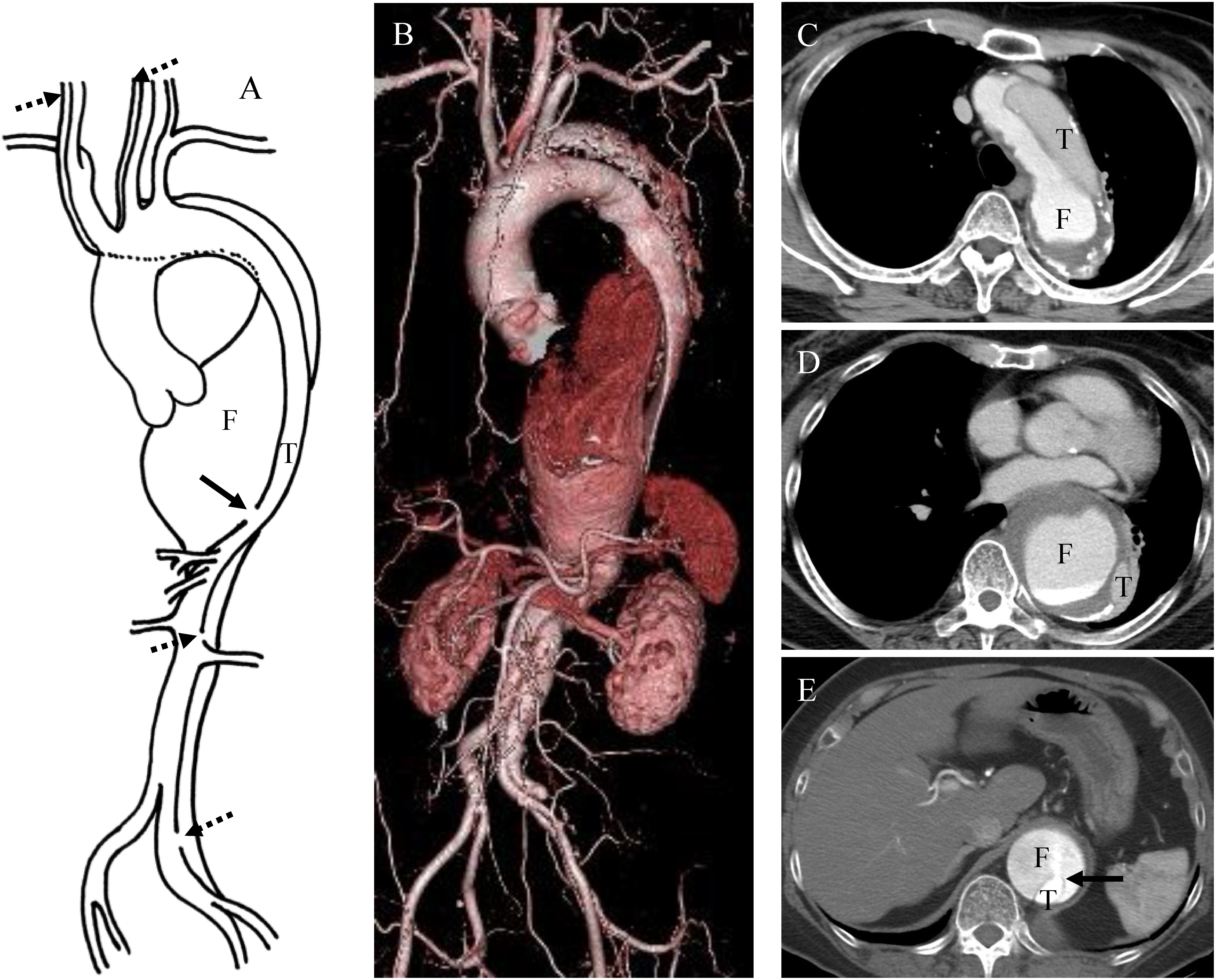
Fig. 1 Preoperative CT angiography findings and schema. Solid black arrow shows the entry of the descending aorta. Broken black arrow shows the re-entry. (**A**) Schema; (**B**) 3-dimensional CT angiography; (**C, D**) CT axial images at late phase; (**E**) CT axial image at arterial phase.

CT: computed tomography; F: false lumen; T: true lumen

A 47-year-old obese woman, who had a history of smoking, presented with untreated hypertension and diabetes mellitus. She was found to have developed acute type-B aortic dissection (type IIIb) consisting of entry at the descending aorta and re-entry of the bilateral common carotid arteries, left renal artery, and left common iliac artery, causing cerebral infarction that further resulted in left hemiplegia. At that time, she had rehabilitation therapy. There was no occlusion of the carotid arteries, even with false lumen patency; thus, no additional intervention was required. She was referred to our hospital and underwent follow-up computed tomography (CT) once a year until the diameter of the descending aorta was observed to have enlarged to 50 mm; thereafter, her visits for follow-up CT were shortened to every 6 months. These CT results, however, now revealed a gradual increase in the diameter of the descending aorta to more than 60 mm with a patent false lumen. Although we have strongly recommended treatment for the dissecting aortic aneurysm, she declined. At 69 years of age, the diameter of the dissecting aortic aneurysm had increased to more than 70 mm, presenting a high probability of aneurysmal rupture, and was already compressing the left atrium. We again explained the necessity of treatment by surgical or endovascular intervention (**Fig. 1**). Finally, she opted for endovascular intervention of the chronic type-B aortic dissection and dissecting aortic aneurysm. We then planned to perform endovascular interventions with occlusion of the entry of the descending aorta and the distal false lumen. At that time, we did not consider occlusion of the re-entry in the carotid artery because of the risk of cerebral arterial emboli. CT angiography revealed sufficient visceral arterial connection between the celiac artery and the superior mesenteric artery, allowing occlusion of the celiac artery. The arteria radicularis magna (Adamkiewicz artery) received its blood supply from the collateral arterial network, including the intercostal artery, which had been occluded via endovascular intervention. Therefore, if she experienced spinal cord injury during the endovascular intervention owing to extensive covering of the aorta, we were prepared to perform immediate spinal fluid drainage. Patient consent for this study was obtained simultaneously with that for the endovascular intervention.

After induction of general anesthesia in a hybrid operating room, we then performed endovascular intervention using a Zenith® Dissection Endovascular Stent (Cook Medical, Inc., LLC, Bloomington, IN, USA). The choice was made to close the entry of the descending aorta and to further embolize the distal false lumen. First, we performed coil embolization of the celiac artery and cannulated the superior mesenteric artery. This was done because the deployment of a stent graft at the level of the superior mesenteric artery was necessitated by substantial covering of the entry of the descending aorta and appropriate embolization of the false lumen in the thoracoabdominal aorta. Because of the difference in terms of the diameter between the true lumen of the aortic arch and the thoracoabdominal aorta, we deployed a stent graft (24-mm diameter, 80-mm length, 5% oversizing of true lumen) in the thoracoabdominal aorta above the superior mesenteric artery, followed by the placement of two stent grafts (both 28-mm diameter, 200-mm length, 5% oversizing of the true lumen) from distal to the left subclavian artery to the thoracoabdominal aorta via the right common femoral artery. We then performed angioplasty of the superior mesenteric artery with a covered stent graft and coil embolization of the false lumen at the level of the branching of the celiac and superior mesenteric arteries. Additionally, we have closed the false lumen of the infrarenal abdominal aorta using the candy-plug technique with a GORE® EXCLUDER® AAA Endoprosthesis aortic extender (26-mm diameter; W. L. Gore & Associates, Inc., Newark, DE, USA) and an AMPLATZER™ Vascular Plug II (16-mm diameter; Abbott Laboratories, Abbott Park, IL, USA) through the left femoral artery. This procedure aims to decrease the flow in the false lumen from the left common iliac artery. The surgery allowed her to maintain her mean blood pressure at ≥90 mmHg and hemoglobin at ≥10 g/dL. The surgery was completed with no complications, and CT angiography showed decreased blood flow in the false lumen with apparent partial thrombosis.

**Figure 2:**
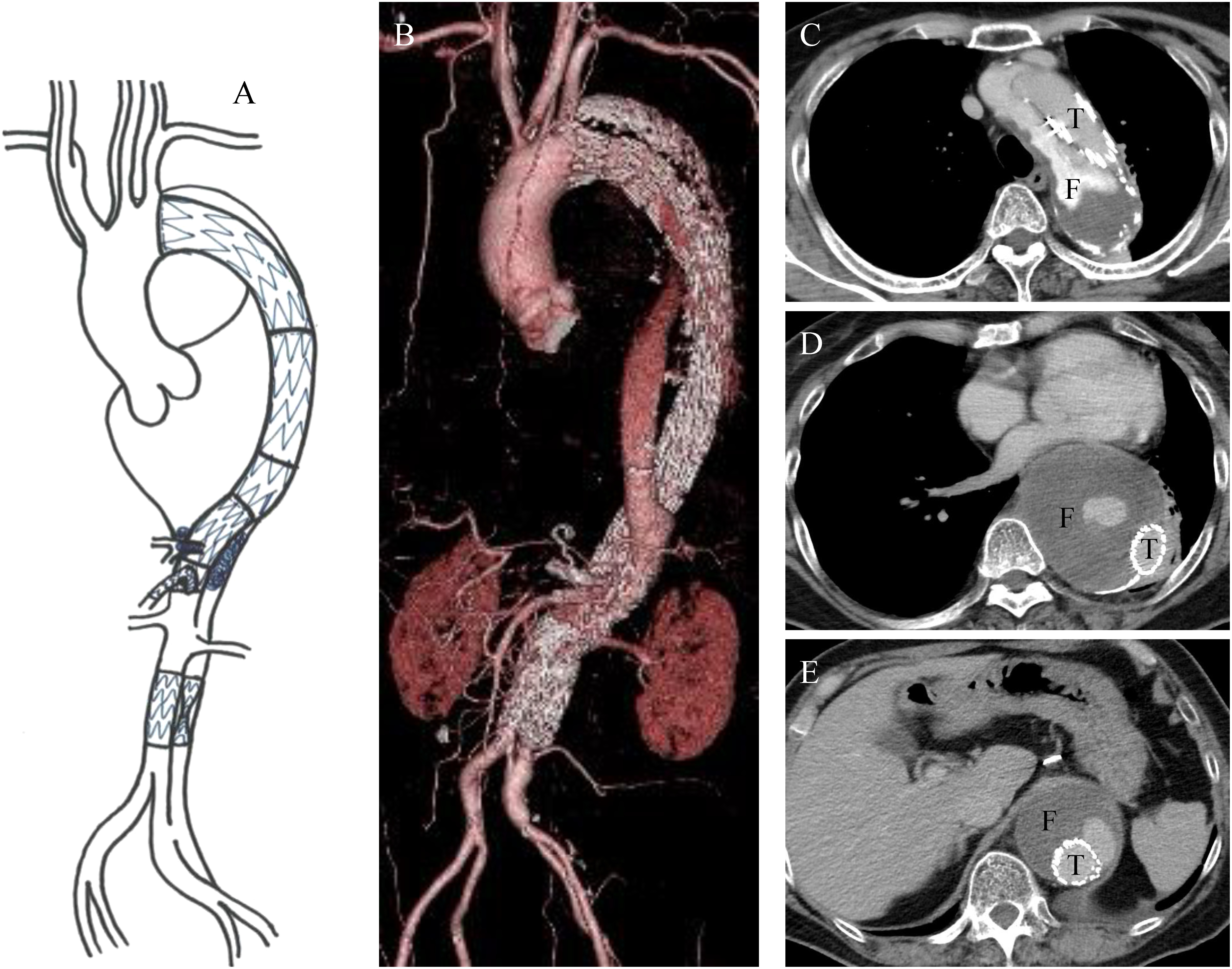
Fig. 2 CT angiography findings and schema after endovascular repair with distal false lumen embolization. (**A**) Schema; (**B**) 3-dimensional CT angiography; (**C**–**E**) CT axial images at late phase.

CT: computed tomography; F: false lumen; T: true lumen

**Figure 3:**
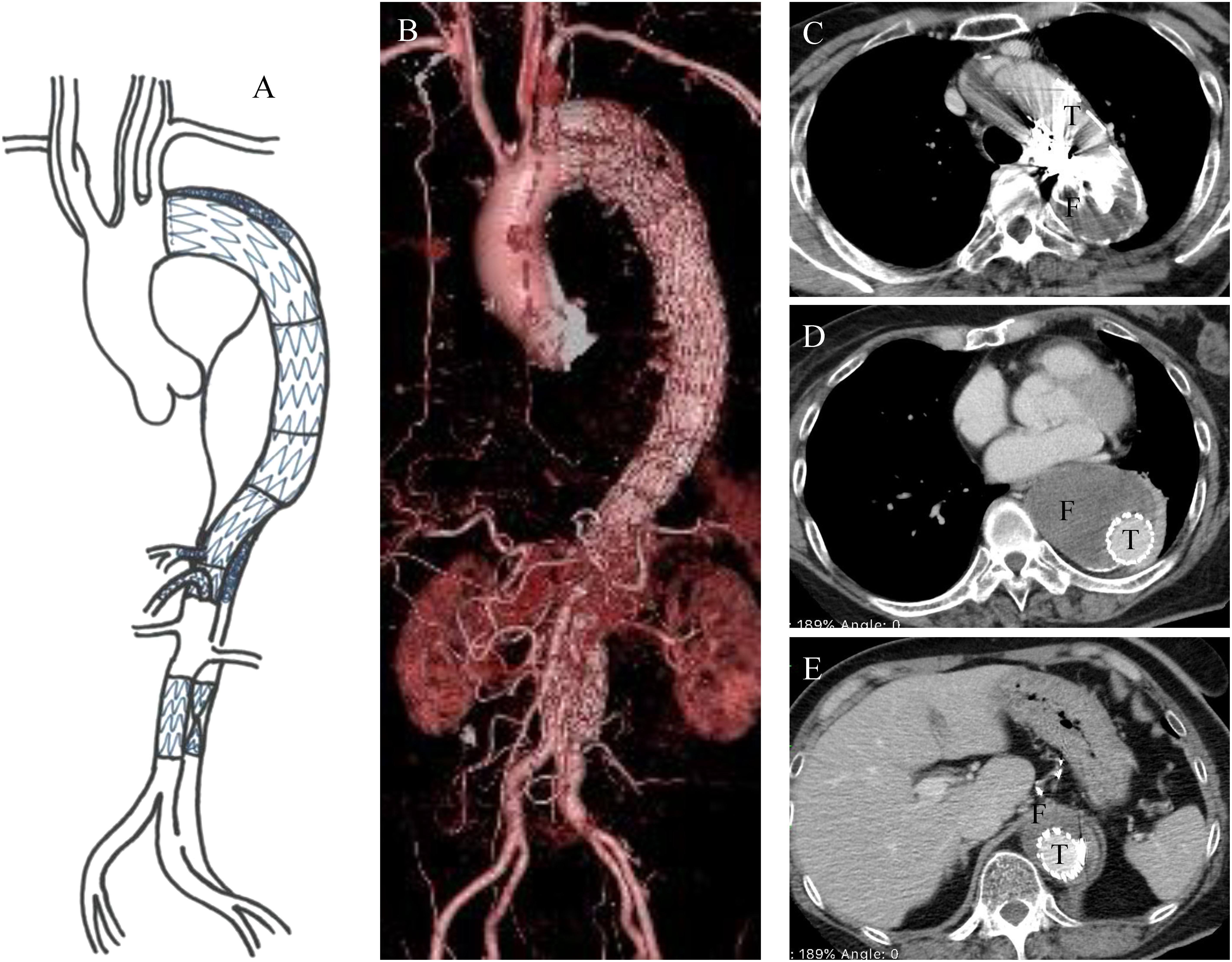
Fig. 3 Postoperative CT angiography findings and schema 1 year after complete false lumen occlusion. (**A**) Schema; (**B**) 3-dimensional CT angiography; (**C**–**E**) CT axial images at late phase.

CT: computed tomography; F: false lumen; T: true lumen

However, follow-up CT angiography revealed further dilation of the false lumen of the descending aorta to more than 80 mm, without complete false lumen thrombosis, due to flow from the re-entry of the carotid artery (**Fig. 2**). Although she rejected open surgical intervention, she did agree to re-intervention in order to prevent this flow. Coil and plug embolization of the aortic arch was thus carried out using an AMPLATZER™ Vascular Plug II (20- and 22-mm diameters) via the false lumen of the right common carotid artery through open puncture of the right common carotid artery, under general anesthesia. Postoperative CT angiography showed complete occlusion of the false lumen at the descending aorta. She then subsequently returned for follow-up visits, with no complications reported. One year after the surgeries, follow-up CT angiography detected appreciable shrinkage of the false lumen of the descending aorta to less than 60 mm with relief of the left atrial compression and no endoleakage (**Fig. 3**).

## Discussion

Surgical intervention remains to be the gold standard treatment for chronic type-B dissecting aortic aneurysms. In a high-volume center, this surgery has provided successful outcomes with low rates of re-intervention and mortality.^4)^ Many recent reports have shown that endovascular interventions for chronic type-B dissecting aortic aneurysm led to good outcomes, with lower rates of morbidity and mortality compared with surgical intervention.^2,3)^ However, in endovascular repair, higher late re-intervention for aortic aneurysmal dilatation compared with surgical repair is an issue.^2,3)^ We experienced a case in which endovascular repair with complete false lumen occlusion provided an excellent outcome, with extreme shrinkage of a dissecting aortic aneurysm that had been present for more than 20 years. No complications were noted.

Patency and degree of patency of the false lumen are indicators of outcome and predictors of the need for re-intervention.^4,5)^ In the chronic phase of dissection, just covering the proximal entry tear and expanding the dissected true lumen can result in unpredictable outcomes, as the residual stable communications between the true and false lumens by a thickened and less mobile dissection flap are responsible for progression of mid- and long-term aneurysmal changes.^6)^ Roselli showed that thoracic endovascular aortic repair for chronic type-B dissection required re-intervention in 22% of patients.^3)^ Additionally, complete false lumen thrombosis occurred in only 13% of patients who had extensive dissection compared with 78% of patients who had dissection limited to the thoracic aorta. The most frequent cause for re-intervention was identified to be aneurysmal dilatation caused by reperfusion from distal re-entry tears left uncovered or newly created, or from visceral branch vessels.^2)^ Therefore, these findings suggest that the main objective of intervention for type-B dissecting aortic aneurysm should be directed at the false lumen, either by promoting thrombosis or by excluding the lumen completely. We also ultimately needed complete occlusion of the false lumen by additional intervention focused on the aortic arch, because the entry and thoracoabdominal re-entry occlusion prevented the curative effects of the dilated aneurysm caused by residual false lumen flow from the carotid artery. Complete false lumen isolation by entry and re-entry occlusion may be a key factor in the endovascular interventions of dissecting aortic aneurysm.

Several techniques, including coil embolization, the candy-plug technique, and the chimney technique, have been reported for occlusion of the false lumen or re-entry.^7–9)^ We then treated the patient’s type-B dissecting aortic aneurysm with combined interventions including the chimney technique, coil and plug embolization, and the candy-plug technique. The optimal embolization technique for false lumen occlusion depends on the anatomical findings. In our case, the false lumen sizes at the aortic arch and at the level of the thoracoabdominal aorta were relatively narrow, which led to good occlusion using the coil and plug methods. However, if the dilated false lumen is larger than the size of the commercial stent graft, whether occluded or not, any occlusion technique may be deemed difficult because of the potential for endoleakage. Additionally, if re-entry is associated with a branching artery, a covered stent with the chimney technique or fenestrated branched technique may be useful for closing the re-entry and substantially restraining the flow of the branching artery. However, required interventions are often cumbersome and difficult when the re-entry involves branching arteries. We therefore need to decide where to occlude the false lumen and which occlusion technique is the most appropriate based on the anatomical findings according to CT angiography.

Spinal cord injury has been identified to be a key major complication of endovascular interventions for extensive thoracic and thoracoabdominal aortic aneurysms. Recent reports showed that 3%–13% of patients who underwent endovascular repair for descending and thoracoabdominal aortic aneurysms suffered a spinal cord injury.^10–13)^ Consequently, neuroprotective interventions (e.g., spinal fluid drainage, maintaining the mean arterial pressure at ≥90 mmHg, methylprednisolone administration) have been reported to prevent or diminish spinal cord injury.^11,12)^ Although spinal fluid drainage is the most effective intervention for this purpose, it has been associated with a >9% incidence of severe or moderate complications (e.g., hematoma, hemorrhage, neurologic deficit, cerebrospinal fluid leakage) that require intervention.^13,14)^ In patients who undergo endovascular repair, however, we can identify the spinal cord injury immediately after the procedure because of the quick arousal from anesthesia. Therefore, even considering its associated risks, we consider spinal fluid drainage immediately in patients who have had spinal cord injury during the endovascular repair. In this present patient, we maintained the mean arterial pressure at ≥90 mmHg and hemoglobin at ≥10 g/dL to prevent iatrogenic spinal cord injury. Fortunately, this patient had no spinal cord injury, which may be due to the development of the collateral network advocated by Etz et al.^15)^ CT angiography showed that the intercostal arteries were occluded by gradual thrombosis of the false lumen before the interventions.

The size of the stent graft is associated with the incidence of adverse events. Retrograde dissection, which is more frequent in thoracic endovascular aortic repair used in dissection than in degenerative aneurysm, is a life-threating complication with high mortality.^16)^ A recent report showed that the risk of retrograde dissection was related to the extent of oversizing of the stent graft in relation to the proximal landing zone, and oversizing the stent graft by more than 9% increased retrograde dissection by 14%.^16)^ We used the recommended 5% oversizing of the true lumen at the deployment site, which may prevent new dissection induced by the stent graft.

Although endovascular interventions with complete false lumen occlusion have a high possibility of remodeling effects for chronic type-B aortic dissection, continual examinations are needed to avoid missing adverse events resulting from residual dissection.

## Conclusion

We reported a successful case of endovascular interventions in a patient with complete false lumen occlusion that resulted in significant shrinkage of a type-B dissecting giant aneurysm that had been present for more than 20 years.
